# Overlap of Subcutaneous Adiposity Across World Health Organization Body Mass Index-for-Age Categories and Anthropometric Correlates of Handgrip Strength in Children Aged 6–12 Years Participating in Organised Club Sport in Buenos Aires, Argentina

**DOI:** 10.3390/children13101358

**Published:** 2026-10-07

**Authors:** Mariano José Giménez, Carlos Abraham Herrera-Amante, Rodrigo Yáñez-Sepúlveda, José Francisco López-Gil

**Affiliations:** 1 Cátedra de Nutrición y Deporte, Escuela de Nutrición, Facultad de Medicina, Universidad de Buenos Aires, Buenos Aires C1121A6B, Argentina; marianogimenez@live.com; 2 Red Iberoamericana de Investigadores en Antropometría Aplicada, 04120 Almería, Spain; carlos.amante@academicos.udg.mx; 3 Laboratorio de Evaluación y Cuidado del Estado Nutricio, Centro Universitario de Tonalá, División de Ciencias de la Salud, Universidad de Guadalajara, Tonalá 45425, Jalisco, Mexico; 4 Faculty of Education and Humanities, School of Sport Sciences, Universidad Andrés Bello, Viña del Mar 2520000, Chile; rodrigo.yanez.s@unab.cl; 5 Department of Sport Sciences, Faculty of Sport and Health Sciences, Fit Generation Research Institute, AD500 Andorra la Vella, Andorra; 6 School of Medicine, Universidad Espíritu Santo, Samborondon 092301, Ecuador; 7 Vicerrectoría de Investigación y Postgrado, Universidad de Los Lagos, Osorno 5290000, Chile

**Keywords:** anthropometry, skinfold thickness, body mass index, hand strength, body composition, child, paediatric obesity, youth sport

## Abstract

**Highlights:**

**What are the main findings?**

• In this convenience sample from two sports clubs, 47.3% of boys and 43.3% of girls were classified as having overweight or obesity; the sum of six skinfolds overlapped by 44–57% between adjacent WHO BMI-for-age categories, and age did not account for this pattern.
• The triceps, supraspinale and abdominal skinfolds ranked children closely against the remaining five sites (*ρ* = 0.901–0.945) and added information beyond BMI-for-age *z*-score; corrected arm girth, unlike relaxed arm girth, remained associated with handgrip strength after adjustment for age, body mass and stature.

**What are the implications of the main findings?**

• A child’s BMI-for-age category conveys limited information about that child’s subcutaneous adiposity; skinfold measurements can complement, but not replace, BMI-based screening.
• Corrected arm girth is a candidate low-cost indicator of the muscular component of the upper arm, whose usefulness relative to handgrip dynamometry requires validation against criterion measures.

**Abstract:**

**Background/Objectives**: Body mass index (BMI) is the most widely used screening tool in paediatric nutritional assessment, but it does not measure body fat directly and conveys no information on muscular development. We aimed to (i) compare the sum of six skinfolds (*Σ*6ST) across World Health Organization (WHO) BMI-for-age categories, accounting for age; (ii) quantify the distributional overlap of *Σ*6ST between categories; (iii) examine how closely each individual skinfold tracks the remaining subcutaneous adiposity; and (iv) examine the associations of upper-limb girths with handgrip strength before and after adjustment for body size. **Methods**: This was a cross-sectional study of a convenience sample of 196 children (129 boys, 67 girls) aged 6–12 years engaged in organised club sport in Morón, Buenos Aires, Argentina. International Society for the Advancement of Kinanthropometry (ISAK)-certified anthropometrists measured six skinfolds and relaxed arm, corrected arm and forearm girths; handgrip strength was assessed with a digital dynamometer. Analyses used Kruskal–Wallis and Brunner–Munzel tests, age-adjusted log-linear models with robust standard errors, kernel-density overlap coefficients (OVLs) with bootstrap confidence intervals, and zero-order and partial Spearman correlations. **Results**: In this sample, 47.3% of boys and 43.3% of girls were classified as having overweight or obesity. Age distributions were similar across BMI categories, and *Σ*6ST differed across categories after age adjustment (*p* < 0.001 in both sexes). Adjacent WHO categories nevertheless overlapped substantially (OVL = 0.44–0.57), and the two halves of the normal-weight range overlapped by up to 0.757. Single skinfolds correlated strongly with the sum of the remaining five (*ρ* = 0.777–0.945) but estimated *Σ*6ST with a median absolute error of 8.5–14.1%. Handgrip strength correlated most strongly with body size (stature in boys, *ρ* = 0.723; body mass in girls, *ρ* = 0.759). After adjustment for age, body mass and stature, it remained associated with corrected arm girth (partial *ρ* = 0.346 and 0.336) but not with relaxed arm girth. **Conclusions**: BMI-for-age categories separate groups on average but carry limited information about an individual child’s subcutaneous adiposity. Skinfolds and corrected arm girth are low-cost complements to BMI whose individual-level value requires validation against criterion methods.

## 1. Introduction

Excess weight is a global epidemic whose high prevalence and rapid growth carry major public health consequences across all age groups and socioeconomic strata. According to the World Health Organization, in 2022, 43% of adults were living with overweight and 16% with obesity, while more than 390 million children and adolescents aged 5–19 years had overweight; prevalence in this age group rose from 8% in 1990 to 20% in 2022 [1]. Pooled analyses of 3663 population-based studies comprising 222 million participants confirm that the combined burden of underweight and obesity increased in the large majority of countries for adults over the same period [2]. The situation is of particular concern in childhood and adolescence, because children with obesity are around five times more likely to be obese in adulthood than their non-obese peers [3], and high childhood BMI is associated with an increased incidence of adult type 2 diabetes, coronary heart disease and several cancers, although its accuracy for predicting any individual morbidity is low [4]. In Latin America and the Caribbean specifically, systematic review evidence documents a high and heterogeneous burden of paediatric overweight and obesity across the region [5].

In clinical practice, children older than two years are routinely evaluated using body mass index (BMI), and those with elevated values are classified as having overweight or obesity [6]. Overweight and obesity are defined as an abnormal or excessive accumulation of body fat; BMI, however, is a mass-for-stature ratio and not a direct measure of adiposity, and it cannot distinguish fat mass from fat-free mass. On the basis of the available evidence, BMI should therefore be regarded primarily as a screening tool. Indeed, BMI is the most widely used screening instrument for identifying obesity in this age group, but its diagnostic performance is markedly asymmetric: pooled sensitivity to detect high adiposity is 0.73 (95% CI 0.67–0.79) against a specificity of 0.93 (0.88–0.96), so that more than a quarter of children with excess body fat are not identified [7]. Skinfold-derived estimates of percent body fat correlate closely with dual-energy X-ray absorptiometry (*r* ≈ 0.85) but are systematically biased at both extremes of the skinfold distribution [8], and BMI *z*-scores are uninformative above the 97th percentile [9]. Among children who already carry excess weight, BMI is a poor predictor of fat mass measured by dual-energy X-ray absorptiometry [10]. A further limitation is interpretative: a child evaluated by BMI alone is followed over time without any parameter describing how the constituent masses are changing.

Several technologies estimate body composition accurately, but their use in the field is constrained by cost, technological complexity and limited portability [11]. Against these limitations, skinfold measurement in paediatric populations is a viable alternative for adding an indicator of subcutaneous adiposity to body-composition assessment, given its accuracy, repeatability, usefulness and low cost [11]. Prediction equations converting paediatric skinfold thicknesses into percent body fat have been available for decades [12], and skinfold data have proved sensitive enough to track secular change in the fatness of young people across more than half a century [13]. Unlike instrumental methods, anthropometry is non-invasive, accessible and portable, which facilitates field application and permits the collection of data for diagnosis, individual follow-up and population-level nutritional surveillance; simple techniques are adequate for many purposes, and simple regional measurements may be of greater value than whole-body values obtained by more sophisticated approaches [11].

In parallel, the World Health Organization recommends that children and adolescents aged 5–17 years accumulate at least 60 min per day of moderate-to-vigorous physical activity, including muscle-strengthening activities [14]. A considerable proportion of this population does not meet these recommendations: pooled data from 298 school-based surveys and 1.6 million students aged 11–17 years placed the global prevalence of insufficient physical activity at 81.0% in 2016 (77.6% of boys, 84.7% of girls) [15], which underlines the importance of assessing not only adiposity but also neuromuscular development, which is itself a powerful marker of current and future health [16,17].

With respect to the monitoring of neuromuscular development, and in line with what has been described for skinfolds, handgrip dynamometry constitutes a relevant functional indicator: it is a simple measurement of maximal voluntary muscle strength that is widely used as a single index of overall muscle strength [18], and normalised handgrip strength discriminates children with elevated cardiometabolic risk with an area under the curve of approximately 0.75–0.77 [19]. Although this value represents function rather than structure, it would be of interest to establish whether it is related to relaxed arm girth, corrected arm girth or forearm girth, and whether any such association persists after accounting for age and body size. Corrected arm girth, which subtracts the contribution of the triceps skinfold from total arm girth, was originally proposed to estimate the muscular component of the limb [20], and handgrip strength and skinfold thickness are themselves core components of validated field-based fitness batteries for youth [21].

Growth and biological maturation add a further layer of complexity in this age range. Between 6 and 12 years, and particularly as puberty approaches, children of the same chronological age differ widely in maturity. Maturation is accompanied by changes in stature, body mass, fat patterning and muscle mass [22,23]. Physical capacities such as muscular strength also change markedly across these years, so part of the variation in anthropometric and functional measures reflects growth and maturity status rather than nutritional status or training [23]. Analyses of adiposity or strength in children spanning this age range should therefore account at least for chronological age, and their results should be interpreted in light of possible differences in maturity.

Given the above, the objectives of this study were: (i) to compare the sum of six skinfolds (*Σ*6ST) across BMI-for-age categories, accounting for age; (ii) to quantify the degree of distributional overlap in *Σ*6ST between those categories; (iii) to examine how closely each individual skinfold tracks the subcutaneous adiposity measured at the remaining sites, and the individual-level error of relying on a single site; and (iv) to examine the associations between upper-limb girths and handgrip strength, before and after adjustment for age and body size. All aims address cross-sectional associations; no diagnostic or predictive accuracy was evaluated.

## 2. Materials and Methods

### 2.1. Study Design

A descriptive, correlational cross-sectional study was conducted. Assessments took place at the premises of Club Deportivo Morón and Club Brisas de Haedo, both located in the municipality of Morón, Province of Buenos Aires, Argentina. The study followed the Strengthening the Reporting of Observational Studies in Epidemiology (STROBE) guidelines [24].

### 2.2. Setting and Ethical Statement

The study protocol was reviewed and approved by the HZGA MYLV Ethics Committee (ACTA-2023-23436469-GDEBA-CECMSALGP; approval date: 11 September 2024). The different sports programmes offered by both clubs were invited to participate in the study. Assessments were conducted at each institution in changing rooms reserved exclusively for data collection during the assessment periods.

All measurements were performed in the presence of a parent or guardian. Written informed consent was obtained from parents or legal guardians, and assent was obtained from the children before participation. Date of birth and biological sex were recorded for age- and sex-specific classification. The study was conducted in accordance with the principles of the Declaration of Helsinki.

### 2.3. Participants

A total of 196 children (129 boys and 67 girls) aged between 6 and 12 years took part in the study. A non-probabilistic convenience sample was recruited from participants in different sports, including football, futsal, roller skating, volleyball, basketball, karate, artistic gymnastics and children’s football (number of participants per discipline: football, 56; futsal, 45; roller skating, 22; volleyball, 12; basketball, 20; karate, 12; artistic gymnastics, 7; children’s football, 22). Data collection was carried out between November and December 2024. The inclusion criteria were membership in one of the two participating clubs; being aged between 6 and 12 years; having written informed consent provided by a parent, guardian, or legal representative; and the child providing assent to participate in the study. Participants were excluded if they had any disease or condition that could affect growth and development, or physical characteristics that prevented any of the required measurements from being performed, such as amputations, physical malformations, Down syndrome, or developmental delays. Participants with incomplete data were excluded from the analysis. As the sample comprised children participating in different sports across two clubs, training volume was not homogeneous among participants. Each sport followed its own training programme and competitive schedule; therefore, participants were not expected to undertake the same volume of training. Nevertheless, as a common criterion, all participants completed a minimum of three training sessions per week. Although participation was open to children from the different sporting activities of both clubs, it was difficult to determine the exact number of children who were invited to participate in the study. We estimate that at least 600 children were invited, of whom 196 participated, corresponding to an estimated participation rate of approximately 32.7%. The sample size was not determined by an a priori calculation; all eligible children whose families consented during the data-collection period were included. With 129 boys and 67 girls, the study had 80% power (two-sided *α* = 0.05) to detect Spearman correlations of about 0.25 in boys and 0.34 in girls. Because some BMI-for-age subgroups contained only 14–16 girls, estimates for girls are less precise, as reflected in their wider confidence intervals.

### 2.4. Assessments

#### 2.4.1. Anthropometric Measurements

All anthropometric measurements were taken by one ISAK Level 3 anthropometrist and two ISAK Level 2 anthropometrists, following the ISAK protocol [25]. Body mass was measured with an Omron HBF-514C electronic scale (Omron Healthcare Co., Ltd., Kyoto, Japan; precision 0.1 kg) and stretch stature with a portable Seca 217 stadiometer (seca GmbH & Co. KG, Hamburg, Germany; range 20–220 cm, precision 0.1 cm). The triceps, subscapular, supraspinale, abdominal, front thigh and medial calf skinfolds were taken with a Harpenden calliper in all children, and arm and forearm girths were measured with an inextensible Lufkin W606PM anthropometric tape (Apex Tool Group, Huntersville, NC, USA). All measurements were taken on the right side of the body under the ISAK protocol. All anthropometric variables were measured at least twice. A third measurement was obtained when the difference between the first two exceeded 5% for skinfolds or 1% for any other variable. When two measurements were taken, the mean value was used for analysis; when three were required, the median value was retained [25]. The intra-observer technical error of measurement (TEM) was as follows: Evaluator 1 (ISAK Level 3), 2.41% for skinfolds and 0.21% for all other measurements; Evaluator 2 (ISAK Level 2), 2.26% for skinfolds and 0.19% for all other measurements; and Evaluator 3 (ISAK Level 2), 2.49% for skinfolds and 0.20% for all other measurements. During several standardization sessions conducted prior to data collection, inter-observer TEM did not exceed 6% for skinfold measurements or 1% for all other anthropometric measurements.

#### 2.4.2. Handgrip Strength

Handgrip strength was assessed using a Takei T.K.K. 5401 Grip-D digital hand dynamometer (Takei Scientific Instruments Co., Ltd., Niigata, Japan; precision 100 g), adjustable to hand size. During data collection, participants remained standing with the arm extended and parallel to the trunk, holding the device and exerting maximal force. An initial familiarization trial was performed, followed by one minute of recovery [26]. The procedure was then repeated twice with each hand. The better of the two attempts performed with the right hand was used in all analyses, including the descriptive comparison between age bands. As anthropometric measurements were taken on the right side of the body in accordance with the ISAK protocol, right-hand grip strength was related to right upper-limb girths, as well as to body mass, stature and age. In addition, the best values obtained with each hand were compared according to hand dominance to determine the proportion of participants who achieved greater handgrip strength with their dominant hand, their non-dominant hand, or identical values with both hands.

#### 2.4.3. Derived Variables

Anthropometric data for body mass and stature were used to compute BMI. Arm, corrected arm and forearm girths were obtained on the right side. Corrected arm girth was derived from the following expression: corrected arm girth (cm) = relaxed arm girth (cm) − π × triceps skinfold (cm) [20]. Corrected arm girth is an anthropometric index of the muscle-plus-bone cross-section of the upper arm; it is not a direct measure of muscle mass [20]. The sum of six skinfolds (*Σ*6ST) comprised the values obtained for the triceps, subscapular, supraspinale, abdominal, thigh and calf sites.

### 2.5. Statistical Analysis

BMI-for-age was classified by sex using the WHO 2007 growth reference for children and adolescents aged 5–19 years [27]. Each child’s BMI was located within the sex-specific percentile channels of the WHO 2007 BMI-for-age tables for the child’s age, and the P3, P85 and P97 curves were used as cut-offs; a child lying exactly on a cut-off was assigned to the higher category. Following WHO terminology, children between P3 and P85 were classified as having normal weight, those between P85 and P97 as having overweight, and those above P97 as having obesity; no participant fell below P3. These three categories constitute the primary analysis. As a secondary analysis, the normal-weight range was divided at P50 (P3–P50 and P50–P85) because this range is broad and potentially heterogeneous. The notation >P97 is used throughout. To verify the classification, BMI-for-age *z*-scores were computed with the WHO 2007 LMS parameters [27]; the P50, P85 and P97 cut-offs correspond to *z*-scores of 0, +1.04 and +1.88. A sensitivity analysis applied the WHO *z*-score cut-offs for overweight (>+1 SD) and obesity (>+2 SD). For descriptive stratification, participants were grouped into two age bands: 6–8 years and 9–12 years.

The analytical strategy was chosen a priori on substantive grounds, not on the basis of normality tests. Skinfold thicknesses, and their sums and girths are strictly positive, right-skewed measurements whose dispersion increases with adiposity; in the present data, a wider interquartile range of *Σ*6ST was observed in boys, ranging from 14.8 mm in the P3–P50 category to 31.2 mm above P97 (Table 3). Selecting a test conditional on a preliminary normality test in the same data also alters the operating characteristics of the subsequent inference [28]. We therefore used rank-based methods that remain valid under unequal dispersion, complemented by parametric models on the log scale with heteroscedasticity-consistent standard errors.

Global differences in *Σ*6ST across BMI-for-age categories were tested with the Kruskal–Wallis test, with effect size *η*^2^[H] = (*H* − *k* + 1)/(*n* − *k*). Under unequal dispersion, the Kruskal–Wallis and Wilcoxon–Mann–Whitney tests assess stochastic dominance rather than differences in medians [29], and they were interpreted accordingly. Pairwise comparisons between categories and between age bands used the Brunner–Munzel test, which assumes neither equal variances nor equal distribution shapes [30]. Multiplicity within each family of comparisons was controlled with Holm’s sequentially rejective procedure [31]. The effect size for pairwise comparisons is the probability of superiority, $\hat{p}$ = P(*X* < *Y*) + 0.5·P(*X* = *Y*). It is the probability that a randomly selected child from the higher category has a larger value than a randomly selected child from the lower category; $\hat{p}$ = 0.5 indicates no stochastic tendency and $\hat{p}$ = 1 complete separation. Dunn’s post hoc tests are also reported for comparability with the originally submitted analysis.

To account for age, *Σ*6ST was log-transformed and modelled by ordinary least squares with BMI-for-age category and decimal age as predictors, separately by sex, using HC3 heteroscedasticity-consistent standard errors [32]. Back-transformed coefficients are ratios of geometric means, and pairwise contrasts were Holm-adjusted. A Welch analysis of variance on log *Σ*6ST was used as a parametric check. The age distribution across BMI-for-age categories was compared with the Kruskal–Wallis test. For the overlap analysis, an age-standardised *Σ*6ST was also computed by removing the within-category age slope estimated in this model, which expresses each child’s value at the mean age of the sample.

The degree of overlap between the *Σ*6ST distributions of two BMI-for-age categories was quantified with the overlap coefficient (OVL), the area shared by the two probability density functions [33]. Densities were estimated separately for each category with a Gaussian kernel and Silverman’s rule-of-thumb bandwidth, 0.9 × min(SD, IQR/1.34) × *n*^−1/5^ [34], evaluated on a common grid of 1024 points spanning both samples. This specification corresponds to the default settings of the R function density. OVL ranges from 0 (no overlap) to 1 (identical distributions). It describes the similarity of two distributions; it is neither an individual misclassification rate nor a measure of the sensitivity or specificity of BMI against an adiposity criterion. Its 95% confidence interval was obtained from 5000 bootstrap resamples drawn with replacement within each category, with the bandwidth re-estimated in every resample, using the 2.5th and 97.5th percentiles. Because OVL depends on the degree of smoothing, a sensitivity analysis multiplied the bandwidth by 0.5, 0.75, 1.5 and 2.

Each skinfold was correlated with the sum of the remaining five sites to avoid part–whole correlation, using Spearman’s *ρ* with percentile bootstrap 95% confidence intervals. Because a rank correlation does not quantify individual error, two further analyses were performed. First, *Σ*6ST was predicted from each single skinfold by log–log regression with leave-one-out cross-validation and smearing back-transformation. Individual error is reported as the median absolute percentage error (MdAPE) and as 95% limits of agreement (LoA) for the ratio of measured to predicted *Σ*6ST [35]. As a reference, the same procedure was applied to BMI-for-age *z*-score plus age. Second, to test whether a single skinfold adds information beyond BMI, the log of the sum of the remaining five sites was regressed on BMI-for-age *z*-score and age. The increase in *R*^2^ after adding the log of the single skinfold was tested with an *F*-test, and the corresponding partial Spearman correlation is also reported.

Associations between right handgrip strength and body mass, stature, age and upper-limb girths were first estimated with zero-order Spearman correlations. To assess whether girths were associated with handgrip strength independently of body size, partial Spearman correlations controlling for age, body mass and stature were computed. In addition, following the allometric approach recommended for youth handgrip strength [36], the log of handgrip strength was regressed on the logs of body mass and stature and on age, and the log of each girth was then added in turn. The girth coefficient in this model is an elasticity, that is, the percentage change in handgrip strength per 1% change in girth. The correlations of corrected and relaxed arm girth with handgrip strength share the same outcome and are therefore dependent. Their difference was estimated with a paired bootstrap for both the zero-order and the partial coefficients.

The originally submitted analyses were carried out in R version 4.4.1 (R Core Team, Vienna, Austria) [37] using the RStudio integrated development environment, version 2023.06.2+561 “Mountain Hydrangea” (Posit, PBC, Boston, MA, USA). The analyses added in revision (Brunner–Munzel tests, log-linear and allometric models, partial correlations, prediction-error analyses and bootstrap procedures) were carried out in Python 3 with SciPy 1.17 and statsmodels 0.15; the kernel density and OVL computations reproduced the R estimates exactly. All bootstrap procedures used 5000 resamples with a fixed random seed (12345). Statistical significance was set at *p* < 0.05 (two-tailed).

## 3. Results

### 3.1. Distribution of BMI-for-Age Categories

In this convenience sample, 47.3% of boys and 43.3% of girls were classified as having overweight or obesity (Table 1). These values describe this sample of club-sport participants and are not prevalence estimates. Age distributions were similar across BMI-for-age categories in boys (*H*(3) = 3.34, *p* = 0.342) and girls (*H*(3) = 1.67, *p* = 0.643), and the proportion of children aged 9–12 years was similar across categories (Table 2). The percentile-based classification agreed with the *z*-score-based classification in 188 of 196 children (95.9%), and all eight discrepancies involved adjacent categories. With the WHO *z*-score cut-offs, the proportion with overweight or obesity was unchanged in boys (47.3%) and was 44.8% in girls.

### 3.2. Sum of Six Skinfolds Across BMI-for-Age Categories

**Figure 1:**
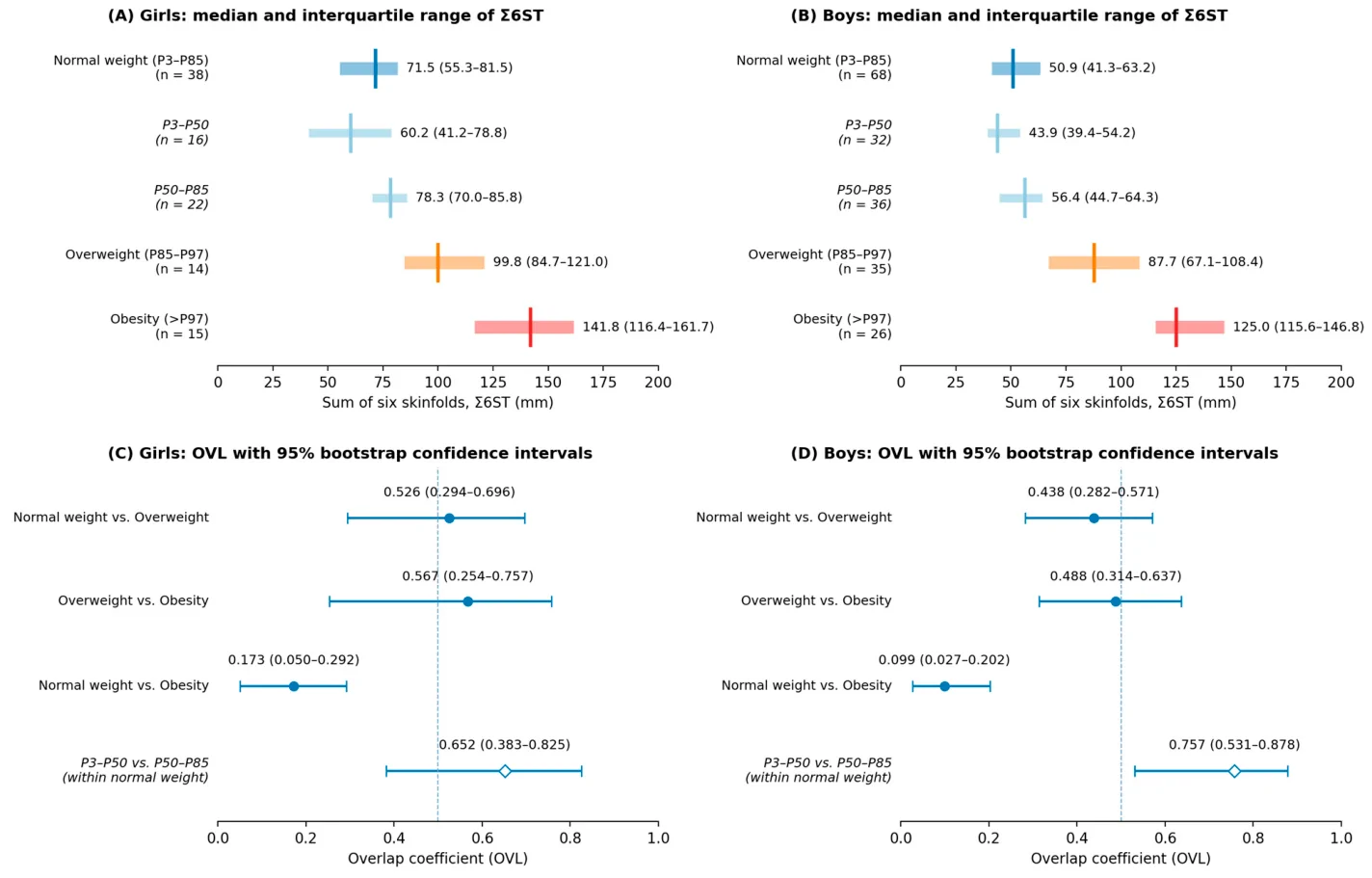
Distribution of the sum of six skinfolds (Σ6ST) by WHO BMI-for-age category in girls and boys. (**A**,**B**) Median (vertical line) and interquartile range (bar) of Σ6ST in each category; the two halves of the normal-weight range (P3–P50 and P50–P85, secondary analysis) are shown in italics. (**C**,**D**) Overlap coefficient (OVL) with 95% bootstrap confidence intervals for pairwise comparisons between WHO categories (filled circles) and between the two halves of the normal-weight range (open diamonds). OVL is the area shared by two kernel density estimates and ranges from 0 (no overlap) to 1 (identical distributions); it is not an individual misclassification rate. The dashed line indicates OVL = 0.5.

In both sexes, *Σ*6ST was higher in each successive BMI-for-age category (Table 3, Figure 1). Among girls, median *Σ*6ST was 71.5 mm in the normal weight group, 99.8 mm in the overweight group and 141.8 mm in the obesity group; among boys, the corresponding values were 50.9, 87.7 and 125.0 mm. Girls showed higher median *Σ*6ST values than boys in every category. The same gradient was present within each age band, although the cells for girls aged 6–8 years contained only 4–10 children (Table 3).

**Table 3:** Sum of six skinfolds (*Σ*6ST, mm) by BMI-for-age category in boys and girls, overall and by age band.

Sex	BMI-for-Age Category	All Ages, *n*	Median (Q1–Q3)	IQR	6–8 Years, *n*	Median (Q1–Q3)	9–12 Years, *n*	Median (Q1–Q3)
Boys	Normal weight (P3–P85)	68	50.9 (41.3–63.2)	21.9	18	40.5 (35.5–48.0)	50	54.4 (43.2–65.8)
	P3–P50 †	32	43.9 (39.4–54.2)	14.8	11	40.4 (35.0–44.9)	21	51.1 (42.1–64.2)
	P50–P85 †	36	56.4 (44.7–64.3)	19.6	7	46.9 (38.8–48.5)	29	59.1 (46.3–66.3)
	Overweight (P85–P97)	35	87.7 (67.1–108.4)	41.3	13	69.6 (58.3–81.4)	22	104.4 (87.8–116.0)
	Obesity (>P97)	26	125.0 (115.6–146.8)	31.2	8	102.8 (95.5–121.5)	18	136.2 (121.0–152.4)
Girls	Normal weight (P3–P85)	38	71.5 (55.3–81.5)	26.2	10	51.5 (41.8–60.8)	28	79.5 (69.5–86.2)
	P3–P50 †	16	60.2 (41.2–78.8)	37.6	5	40.4 (38.4–48.9)	11	71.0 (60.2–82.4)
	P50–P85 †	22	78.3 (70.0–85.8)	15.8	5	62.8 (54.1–70.7)	17	80.6 (74.9–92.3)
	Overweight (P85–P97)	14	99.8 (84.7–121.0)	36.3	4	78.8 (72.3–85.8)	10	111.2 (99.7–133.4)
	Obesity (>P97)	15	141.8 (116.4–161.7)	45.3	4	131.6 (122.1–143.4)	11	150.6 (116.4–164.8)

Q1, first quartile; Q3, third quartile; IQR, interquartile range. † Secondary analysis: halves of the normal-weight range.

### 3.3. Sum of Six Skinfolds by Age Band

When the sum of six skinfold thicknesses (*Σ*6ST) was compared between age bands, higher values were observed among participants aged 9–12 years than among those aged 6–8 years in both girls and boys (Table 4).

Among girls, *Σ*6ST was higher in those aged 9–12 years (median 90.9 mm; Q1–Q3 78.6–117.1) than in those aged 6–8 years (67.1 mm; 50.2–93.0; Brunner–Munzel *p* = 0.015). The probability of superiority was $\hat{p}$ = 0.71 (95% CI 0.54–0.88): in 71% of random pairs, the older girl had the larger *Σ*6ST.

Similarly, among boys, *Σ*6ST was higher in those aged 9–12 years (74.8 mm; 52.8–115.1) than in those aged 6–8 years (58.3 mm; 41.0–83.4; *p* = 0.005), with $\hat{p}$ = 0.65 (95% CI 0.55–0.76).

These are moderate effects: in roughly two of every three pairs, the older child had the larger *Σ*6ST. The effect was somewhat larger in girls, although the confidence intervals overlapped. In the age-adjusted models (Section 3.5), *Σ*6ST was about 8% higher per year of age in both sexes, independent of BMI-for-age category.

### 3.4. Upper-Limb Girths and Handgrip Strength by Age Band

In both sexes, upper-limb girths and handgrip strength were higher in the 9–12-year age group than in the 6–8-year age group (all Brunner–Munzel *p* ≤ 0.006). In boys, median right handgrip strength increased from 11.0 kg at 6–8 years to 15.8 kg at 9–12 years, while in girls, it increased from 11.2 to 15.3 kg; the corresponding probabilities of superiority were 0.86 and 0.84. Relaxed arm, corrected arm, and forearm girths also showed higher median values in the older age group in both sexes (Table 5).

### 3.5. Differences in Σ6ST Between BMI-for-Age Categories

*Σ*6ST differed across WHO BMI-for-age categories in both sexes (girls: *H*(2) = 38.34, *p* < 0.001, *η*^2^[H] = 0.568; boys: *H*(2) = 79.40, *p* < 0.001, *η*^2^[H] = 0.614). With the normal-weight range divided at P50, the results were similar (girls: *H*(3) = 40.89, *η*^2^[H] = 0.601; boys: *H*(3) = 81.75, *η*^2^[H] = 0.630; both *p* < 0.001). These are large effects, with BMI-for-age category accounting for roughly 60% of the rank variation in *Σ*6ST. In pairwise Brunner–Munzel comparisons, every pair of WHO categories differed after Holm adjustment (Table 6). The probability of superiority was 0.86–0.88 for normal weight versus overweight and 0.80–0.88 for overweight versus obesity. A randomly selected child with overweight therefore had a larger *Σ*6ST than a randomly selected child with normal weight in 86–88% of pairs, but not in the remaining 12–14%.

Within the normal-weight range, children in the P50–P85 half had higher *Σ*6ST than those in the P3–P50 half (girls: $\hat{p}$ = 0.72, Holm-adjusted *p* = 0.016; boys: $\hat{p}$ = 0.66, adjusted *p* = 0.019). Dunn’s test did not reach significance for this contrast (adjusted *p* = 0.118 and 0.125). Adjustment for age did not change these conclusions. In the age-adjusted log-linear models, BMI-for-age category remained strongly associated with *Σ*6ST (girls: *F*(2,63) = 47.36; boys: *F*(2,125) = 158.03; both *p* < 0.001). Relative to normal weight, the geometric mean of *Σ*6ST in girls was 1.53 times higher with overweight (95% CI 1.31–1.78) and 2.10 times higher with obesity (1.80–2.44). In boys, the corresponding ratios were 1.72 (1.56–1.90) and 2.58 (2.31–2.88). Each additional year of age was associated with about 8% higher *Σ*6ST (girls: ratio 1.077, 95% CI 1.039–1.116; boys: 1.080, 1.055–1.106). A Welch analysis of variance on log *Σ*6ST led to the same conclusions (both *p* < 0.001).

### 3.6. Distributional Overlap of Σ6ST Between BMI-for-Age Categories

Although BMI-for-age categories differed clearly at the group level, their *Σ*6ST distributions were not separate (Table 6, Figure 1).

Among girls, OVL was 0.526 (95% CI 0.294–0.696) between normal weight and overweight and 0.567 (0.254–0.757) between overweight and obesity, and fell to 0.173 (0.050–0.292) between normal weight and obesity. Within the normal-weight range, the P3–P50 and P50–P85 halves overlapped by 0.652 (0.383–0.825).

Among boys, the corresponding values were 0.438 (0.282–0.571), 0.488 (0.314–0.637) and 0.099 (0.027–0.202), and the two halves of the normal-weight range overlapped by 0.757 (0.531–0.878).

Overlap depended on smoothing, but its pattern did not. With bandwidth multipliers from 0.5 to 2, OVL between adjacent WHO categories ranged from 0.42 to 0.55 in boys and from 0.49 to 0.65 in girls, and OVL between normal weight and obesity from 0.07 to 0.17 and from 0.13 to 0.27, respectively. After standardising *Σ*6ST to the mean age of the sample, overlap between adjacent WHO categories was 0.31–0.41 in boys and 0.40–0.52 in girls, and overlap within the normal-weight range remained 0.71 in boys and 0.61 in girls (Table 6). Age therefore contributed to, but did not explain, the overlap.

### 3.7. Association of Individual Skinfolds with the Remaining Subcutaneous Adiposity

Because a six-site assessment is not always feasible, we examined how closely each single skinfold tracked the adiposity measured at the other sites, how large the individual error would be if *Σ*6ST were estimated from that site alone, and whether the site added information beyond BMI.

Each skinfold was correlated with the sum of the remaining five sites rather than with the full *Σ*6ST, to avoid part–whole correlation (Table 7).

All six sites correlated strongly with the sum of the remaining five in both sexes (all *p* < 0.001). In girls, *ρ* ranged from 0.777 for the thigh (95% CI 0.622–0.885) to 0.916 for the triceps (0.851–0.948), with the abdominal (0.913), subscapular (0.901) and supraspinale (0.901) sites close behind. In boys, *ρ* ranged from 0.867 for the thigh (0.796–0.909) to 0.945 for the supraspinale (0.915–0.961), followed by the triceps (0.935), abdominal (0.934) and subscapular (0.931) sites.

Strong rank correlations did not translate into small individual errors. When *Σ*6ST was predicted from a single site, the median absolute error was 8.5–14.1% in boys and 9.1–11.4% in girls, and the 95% limits of agreement for the ratio of measured to predicted *Σ*6ST were about 0.74–1.32 for the triceps, supraspinale and abdominal sites (Table 7). For comparison, BMI-for-age *z*-score and age predicted *Σ*6ST with a median absolute error of 14.9% in boys and 11.5% in girls. Every single site added information beyond BMI-for-age *z*-score and age (all *p* < 0.001). The increase in explained variance was 0.11–0.17 in boys and 0.04–0.08 in girls, and partial correlations ranged from 0.70 to 0.82 in boys and from 0.40 to 0.65 in girls.

Statistically, the supraspinale site showed the strongest correlation in boys and the triceps in girls, and the triceps, supraspinale and abdominal sites had the smallest prediction errors in both sexes. Among these, the triceps and abdominal sites are also easy to locate and require minimal body exposure; this is a practical consideration rather than a statistical one.

### 3.8. Anthropometric Correlates of Handgrip Strength

Regarding hand dominance, most participants were right-handed, including 58 girls (86.6%) and 106 boys (82.2%), whereas 9 girls (13.4%) and 23 boys (17.8%) were left-handed. Overall, 119 participants (60.7%) achieved a higher handgrip strength value with their dominant hand, 74 (37.8%) achieved a higher value with their non-dominant hand, and 3 (1.5%) obtained identical values with both hands. This pattern was similar in both sexes. Among girls, 41 (61.2%) achieved a higher value with the dominant hand, 25 (37.3%) with the non-dominant hand, and 1 (1.5%) obtained identical values with both hands. Among boys, the corresponding values were 78 (60.5%), 49 (38.0%), and 2 (1.6%), respectively. Thus, although right-hand dominance predominated in both sexes, the dominant hand did not invariably correspond to the hand producing the highest grip strength, with more than one-third of both girls and boys achieving a higher value with their non-dominant hand. In both sexes, right-hand grip strength was positively and significantly correlated with all anthropometric variables and age (*p* < 0.001) (Table 8).

In girls, handgrip strength correlated most strongly with body mass (*ρ* = 0.759) and corrected arm girth (*ρ* = 0.747), followed by forearm girth (*ρ* = 0.701), stature (*ρ* = 0.683), relaxed arm girth (*ρ* = 0.643), and age (*ρ* = 0.606). In boys, the strongest correlations were with stature (*ρ* = 0.723) and body mass (*ρ* = 0.654), followed by age (*ρ* = 0.611), corrected arm girth (*ρ* = 0.599), forearm girth (*ρ* = 0.576), and relaxed arm girth (*ρ* = 0.453). The correlation with handgrip strength was higher for corrected than for relaxed arm girth, 0.146 higher (95% CI 0.073–0.225) in boys and 0.104 higher (0.020–0.199) in girls.

After adjustment for age, body mass and stature, corrected arm girth remained associated with handgrip strength (partial *ρ* = 0.346, 95% CI 0.153–0.505 in boys; 0.336, 0.081–0.626 in girls). Relaxed arm girth showed no adjusted association (−0.017, −0.216 to 0.189; and 0.078, −0.182 to 0.361). Forearm girth showed an intermediate association (0.239, 0.062–0.409; and 0.214, −0.054 to 0.460). In the allometric models, body size and age explained 58% of the variance in log handgrip strength in boys and 70% in girls. Adding log corrected arm girth increased *R*^2^ by 0.058 in boys (elasticity 1.25, 95% CI 0.66–1.84, *p* < 0.001) and by 0.032 in girls (0.88, −0.15 to 1.92, *p* = 0.095). The adjusted difference between corrected and relaxed arm girth was 0.364 (0.169–0.542) in boys and 0.258 (0.059–0.475) in girls. Corrected arm girth therefore carried information on handgrip strength beyond body size in boys; in girls, the evidence was consistent in direction but less precise.

## 4. Discussion

This study examined subcutaneous adiposity across BMI-for-age categories and the anthropometric correlates of handgrip strength in 196 children aged 6–12 years participating in organised club sport. Three findings stand out. First, 47.3% of boys and 43.3% of girls in this sample were classified as having overweight or obesity. Second, *Σ*6ST differed markedly across WHO BMI-for-age categories, independently of age, yet the distributions of adjacent categories overlapped by 44–57%, and those of the two halves of the normal-weight range by up to 75.7%. Third, single skinfolds ranked children closely against the remaining sites but carried substantial individual error. Corrected arm girth, unlike relaxed arm girth, remained associated with handgrip strength after adjustment for age, body mass and stature, most clearly in boys.

### 4.1. Weight Status in a Physically Active Paediatric Sample

The proportion of children with overweight or obesity in this sample (47.3% of boys, 43.3% of girls) is a characteristic of a convenience sample from two clubs, not a prevalence estimate. It is nonetheless notable because participation in organised sport is often assumed to be protective. Excess body weight has also been described among young athletes elsewhere. In 40,077 pre-participation examinations of Estonian young athletes, overweight plus obesity affected approximately 19–22% of boys and 12–15% of girls, with no significant temporal trend between 2010 and 2024 [38]. The frequencies observed here are descriptively higher, but differences in sampling, age range, sports and population prevent a direct comparison. Regional context also matters: the burden of paediatric overweight and obesity in Latin America is already high and heterogeneous [5]. Two non-exclusive explanations deserve consideration. Sport participation may be insufficient to offset the obesogenic environment when weekly training volume is modest, and BMI may overestimate adiposity in children with greater lean mass. The present data bear only indirectly on the second explanation. Higher BMI-for-age categories were accompanied, on average, by higher *Σ*6ST, but without a reference measure of body composition we cannot establish to what extent differences in BMI reflect adiposity rather than fat-free components.

### 4.2. Group-Level Separation and Distributional Overlap

A large Kruskal–Wallis effect and substantial overlap between adjacent categories are not contradictory. Tests and effect sizes describe differences between group distributions, whereas OVL describes how much of those distributions is shared. In girls, the *Σ*6ST distributions of adjacent WHO categories shared 53–57% of their area, and in boys, it is 44–49%. Within the normal-weight range, the two halves shared 65.2% in girls and 75.7% in boys, even though Brunner–Munzel tests detected a stochastic difference between them. Only the comparison between normal weight and obesity showed limited overlap (10% in boys and 17% in girls). Age distributions were similar across categories, and the pattern persisted after age standardisation, so differences in age composition are unlikely to explain these findings. The probabilities of superiority convey the same message for pairs of children. A child with overweight had a larger *Σ*6ST than a child with normal weight in 86–88% of random pairs, which leaves a non-trivial fraction of pairs in the opposite direction.

These overlaps should not be read as misclassification rates. OVL quantifies the area shared by two estimated distributions; it is neither the probability that a BMI-based inference about a given child is wrong nor a measure of sensitivity or specificity against an adiposity criterion. Such measures would require a prespecified criterion and a reference method. What the data do show is that knowing a child’s BMI-for-age category conveys limited information about where that child lies in the *Σ*6ST distribution. This interpretation is consistent with meta-analytic evidence that BMI has high specificity (0.93) but lower sensitivity (0.73) for detecting excess adiposity in children [7]. It is also consistent with evidence that BMI z-scores become less informative above the 97th percentile [9], and with evidence of the limited ability of BMI to predict fat mass measured by dual-energy X-ray absorptiometry among 663 children with overweight or obesity [10].

However, these findings should not be interpreted as indicating that skinfold measurements are universally superior to BMI as predictors of health risk. In NHANES, lipid levels showed similar associations with BMI and skinfold-derived percentage body fat, with only small differences observed for blood pressure [8]. The rationale for including skinfold measurements is therefore not that they necessarily provide better prediction of cardiovascular risk, but that they provide information on subcutaneous adiposity that BMI, as a mass-for-stature index, cannot directly quantify. In this context, skinfold measurements should be viewed as complementary to, rather than a replacement for, BMI in paediatric nutritional assessment.

Reporting OVL alongside conventional tests is therefore useful in paediatric anthropometry, because it separates group-level statistical differences from the extent to which distributions remain shared [33].

### 4.3. Single Skinfolds as Indicators of Overall Subcutaneous Adiposity

All six sites correlated strongly with the sum of the remaining five, and the triceps, supraspinale and abdominal sites were among the sites with the highest coefficients and had the smallest prediction errors in both sexes. These results indicate strong rank-order association, not interchangeability. A single site ranked children as much as the multisite sum did, but estimating an individual child’s *Σ*6ST from one site carried wide 95% limits of agreement: measured values ranged from about 26% below to 32% above the predicted value. Single sites nevertheless added information beyond BMI-for-age *z*-score, which supports measuring at least one skinfold alongside BMI when the full protocol is not feasible. The choice among the best sites can then rest on practical grounds. The triceps and abdominal sites require minimal body exposure, and the triceps has the largest body of paediatric reference data [39,40] and is already used to calculate corrected arm girth [20]. Published paediatric equations based on the triceps and subscapular sites rest on the same premise of site representativeness [12]. The front thigh performed worst in both sexes, particularly in girls (*ρ* = 0.777; 95% CI 0.622–0.885). This is consistent with sex differences in the development of lower-limb fat during the school years [39,40] and argues against relying on a single lower-limb site. Whether a one- or two-site protocol can substitute for *Σ*6ST at the individual level requires a formal agreement study against a criterion method [35].

### 4.4. Upper-Limb Girths and Handgrip Strength

Handgrip strength was positively associated with all the anthropometric dimensions assessed, and most strongly with body size. Stature and body mass showed the highest zero-order correlations in boys, whereas in girls, body mass showed the highest zero-order correlations, closely followed by corrected arm girth; this is expected in a sample spanning six years of growth.

Corrected arm girth correlated more strongly with handgrip strength than relaxed arm girth, and the paired bootstrap supported this difference in both sexes. The adjusted analyses are more informative. Once age, body mass and stature were controlled, relaxed arm girth was no longer associated with handgrip strength, whereas corrected arm girth still was. This pattern is consistent with the rationale of corrected arm girth, which removes the contribution of subcutaneous fat to approximate the muscle-plus-bone component of the arm [20].

In the allometric models, corrected arm girth added 5.8% of explained variance in boys, with an elasticity of 1.25, but only 3.2% in girls, with a confidence interval that included zero. The smaller number of girls limits precision, so the result in girls should be regarded as inconclusive rather than negative. Forearm girth showed a weaker adjusted association than corrected arm girth. These findings describe associations only. Corrected arm girth is not a substitute for handgrip dynamometry, and it was not validated here against a criterion measure of muscle mass.

This interpretation is supported by studies using criterion methods showing that fat-free mass and lean soft tissue mass, measured by dual-energy X-ray absorptiometry (DXA), are associated with handgrip strength in children and adolescents (in a cohort living with HIV) [41]. Likewise, handgrip strength expressed relative to BMI has been shown to be more closely associated with physical fitness than BMI itself in a physically active paediatric population [42]. Sport-specific data further indicate that differences in handgrip strength between young athletes and non-athletes emerge progressively across the developmental years [43], which is relevant to a sample recruited entirely from organised sport.

### 4.5. Practical Implications

Three cautious implications follow for school and club settings. First, BMI-for-age remains a useful screening instrument, but it conveys limited information about an individual child’s subcutaneous adiposity and should not be presented to families or coaches as a direct statement about a child’s fatness. Second, measuring one or two skinfolds requires only a calliper and trained staff, and in this sample, single sites added information beyond BMI. Whether this improves decisions about individual children, or longitudinal monitoring in which BMI change alone cannot distinguish fat gain from lean gain [11], has not been tested. Third, corrected arm girth may offer a low-cost indicator of the muscular component of the upper arm. Muscular fitness is increasingly recognised as a marker of cardiometabolic risk in youth [16,17,19] and, in a cohort of more than one million adolescents, of premature mortality in later life [44]; it is included in consensus surveillance batteries [21,45], for which large-scale European normative values exist [46]. None of these anthropometric measures was evaluated here for diagnostic accuracy, so they should be regarded as candidate complements to BMI rather than as screening tools in their own right.

### 4.6. Strengths and Limitations

The main strengths of the study are the use of ISAK Level 2 and Level 3 anthropometrists throughout, the full six-site skinfold protocol, the deliberate avoidance of part–whole correlation when evaluating individual skinfolds, the use of overlap coefficients with bootstrap confidence intervals and bandwidth sensitivity analysis, and the combination of rank-based methods valid under unequal dispersion with age- and body-size-adjusted models.

The limitations can be grouped by their consequences for inference. Design: The study is cross-sectional, so it supports associations only, without causal, temporal or diagnostic inference. Maturation: Biological maturation was not assessed by any method (Tanner stage, age at peak height velocity, or the maturity offset predicted from anthropometry [22]). This matters in a sample spanning 6–12 years because stature, body mass, fat patterning and strength all depend on maturity [23]. Adjustment for chronological age does not remove differences in maturity between children of the same age, so residual confounding by maturation is possible in both the adiposity and the handgrip analyses. Sampling and precision: The sample was a non-probabilistic convenience sample from two clubs in a single municipality, with heterogeneous sports and training volumes, and its size was not determined a priori. Boys outnumbered girls (129 vs. 67), some BMI-for-age strata contained only 14–16 girls, and the strata of girls aged 6–8 years were very small; estimates for girls are therefore less precise, as the wide confidence intervals in Table 6, Table 7 and Table 8 and Figure 1 show. Measurement: Skinfold thickness reflects subcutaneous fat only; no criterion method such as DXA, air-displacement plethysmography or a four-compartment model was available [11], and corrected arm girth was not validated against a measure of muscle mass. Anthropometry was measured on the right side only, although population-based data show systematic between-side differences in handgrip strength [47], and neither training volume nor dietary intake was quantified. Classification: The choice of growth reference may substantially affect the classification of children into BMI categories [48], and the division of the normal-weight range at P50 is a secondary, study-specific subdivision.

### 4.7. Future Directions

Four extensions follow from these data: (i) repeating the adjusted and allometric handgrip analyses with an assessment of maturity status [22,36]; (ii) agreement analyses (Bland–Altman limits of agreement, not correlation [35]) testing whether a one- or two-site protocol can substitute for *Σ*6ST and for a criterion method; (iii) external validation of the overlap findings in a larger, probabilistically sampled cohort with balanced sex representation, including diagnostic-accuracy analyses against a prespecified adiposity criterion; and (iv) prospective follow-up to determine whether children whose skinfold profile is discordant with their BMI category differ in cardiometabolic trajectory.

## 5. Conclusions

In this sample of children aged 6–12 years participating in organised club sport, the sum of six skinfolds was higher in higher WHO BMI-for-age categories, independent of age. Yet the distributions of adjacent categories overlapped by 44–57%, and those of the two halves of the normal-weight range by up to 75.7%. BMI-for-age categories therefore separate groups on average but carry limited information about an individual child’s subcutaneous adiposity. Single skinfolds, particularly the triceps, supraspinale and abdominal sites, were strongly associated with the adiposity measured at the remaining sites and added information beyond BMI, but they did not estimate the six-site sum precisely enough to replace it for individual children. Handgrip strength was most strongly associated with body size. After adjustment for age, body mass and stature, corrected arm girth remained associated with handgrip strength, whereas relaxed arm girth did not. These cross-sectional associations support skinfolds and corrected arm girth as low-cost candidate complements to BMI, whose value for individual assessment requires validation against criterion methods.

## Figures and Tables

**Table 1:** Distribution of boys and girls aged 6–12 years by BMI-for-age category.

Group	*n*	Normal Weight		Overweight	Obesity	Overweight + Obesity
		P3–P50	P50–P85	P85–P97	>P97	
Boys	129	32 (24.8%)	36 (27.9%)	35 (27.1%)	26 (20.2%)	47.3%
Girls	67	16 (23.9%)	22 (32.8%)	14 (20.9%)	15 (22.4%)	43.3%

Values are *n* (%), stratified by sex. Normal weight, P3–P85; overweight, P85–P97; obesity, >P97 (WHO 2007 BMI-for-age reference). Percentages describe this convenience sample and are not prevalence estimates.

**Table 2:** Age distribution across BMI-for-age categories in boys and girls.

Sex	BMI-for-Age Category	*n*	Age, Years, Median (Q1–Q3)	6–8 Years, *n* (%)	9–12 Years, *n* (%)
Boys	P3–P50	32	11.0 (8.7–12.3)	11 (34.4%)	21 (65.6%)
	P50–P85	36	10.7 (9.5–11.6)	7 (19.4%)	29 (80.6%)
	P85–P97 (overweight)	35	9.9 (8.6–11.1)	13 (37.1%)	22 (62.9%)
	>P97 (obesity)	26	10.2 (8.6–11.1)	8 (30.8%)	18 (69.2%)
	Test across categories		*H*(3) = 3.34; *p* = 0.342		
Girls	P3–P50	16	10.7 (8.4–11.1)	5 (31.2%)	11 (68.8%)
	P50–P85	22	10.5 (9.4–11.3)	5 (22.7%)	17 (77.3%)
	P85–P97 (overweight)	14	9.6 (8.8–11.0)	4 (28.6%)	10 (71.4%)
	>P97 (obesity)	15	9.4 (8.9–10.7)	4 (26.7%)	11 (73.3%)
	Test across categories		*H*(3) = 1.67; *p* = 0.643		

Q1, first quartile; Q3, third quartile. Age was compared across categories with the Kruskal–Wallis test (*H*).

**Table 4:** Median and dispersion measures of the sum of six skinfolds (*Σ*6ST, mm) in boys and girls aged 6–12 years, stratified by age band.

Group	*n*	Median (mm)	Q1–Q3 (IQR) (mm)	P10 (mm)	P90 (mm)	$\hat{p}$ **(95% CI)**
Boys 6–8 years	39	58.3	41.0–83.4 (42.4)	35.2	100.9	
Boys 9–12 years	90	74.8	52.8–115.1 (62.3)	42.1	133.0	0.65 (0.55–0.76)
Girls 6–8 years	18	67.1	50.2–93.0 (42.8)	39.8	129.2	
Girls 9–12 years	49	90.9	78.6–117.1 (38.5)	66.1	158.5	0.71 (0.54–0.88)

P10, 10th percentile; P90, 90th percentile; $\hat{p}$, probability that a randomly selected child aged 9–12 years has a larger *Σ*6ST than a randomly selected child aged 6–8 years (Brunner–Munzel).

**Table 5:** Relaxed arm, corrected arm and forearm girths and right handgrip strength in boys and girls, stratified by age band.

Sex and Age Band	Variable	Median	P25	P75	IQR
Boys 6–8 years	Relaxed arm girth (cm)	19.5	18.3	21.4	3.1
	Corrected arm girth (cm)	16.6	15.7	17.5	1.8
	Forearm girth (cm)	19.1	17.9	20.4	2.5
	Right handgrip strength (kg)	11.0	9.3	12.8	3.5
Boys 9–12 years	Relaxed arm girth (cm)	21.8	20.0	24.3	4.3
	Corrected arm girth (cm)	17.8	16.8	19.5	2.7
	Forearm girth (cm)	20.4	19.5	21.9	2.4
	Right handgrip strength (kg)	15.8	13.4	18.6	5.2
Girls 6–8 years	Relaxed arm girth (cm)	19.1	17.1	22.6	5.5
	Corrected arm girth (cm)	15.1	14.0	17.4	3.4
	Forearm girth (cm)	17.9	16.8	19.8	3.0
	Right handgrip strength (kg)	11.2	8.2	13.2	5.0
Girls 9–12 years	Relaxed arm girth (cm)	22.5	20.4	25.2	4.8
	Corrected arm girth (cm)	17.7	16.4	19.8	3.4
	Forearm girth (cm)	20.2	19.0	22.0	3.0
	Right handgrip strength (kg)	15.3	12.7	18.1	5.4

P25, 25th percentile; P75, 75th percentile; IQR, interquartile range. Handgrip strength is the better of two right-hand attempts.

**Table 6:** Pairwise comparisons and distributional overlap of the sum of six skinfolds (*Σ*6ST) between BMI-for-age categories in boys and girls.

Comparison	$\hat{p}$ ** (95% CI)**	Brunner–Munzel *p*_adj_	Dunn *p*_adj_	Age-Adjusted GM Ratio (95% CI)	OVL (95% CI)	Age-Standardised OVL (95% CI)
**Girls**						
Normal weight vs. Overweight	0.86 (0.73–0.98)	<0.001	<0.001	1.53 (1.31–1.78)	0.526 (0.294–0.696)	0.403 (0.184–0.602)
Normal weight vs. Obesity	0.99 (0.96–1.00)	<0.001	<0.001	2.10 (1.80–2.44)	0.173 (0.050–0.292)	0.167 (0.043–0.272)
Overweight vs. Obesity	0.80 (0.62–0.98)	0.003	0.059	1.37 (1.15–1.63)	0.567 (0.254–0.757)	0.520 (0.203–0.705)
P3–P50 vs. P50–P85 †	0.72 (0.54–0.90)	0.016	0.118	1.32 (1.12–1.55)	0.652 (0.383–0.825)	0.608 (0.343–0.781)
**Boys**						
Normal weight vs. Overweight	0.88 (0.81–0.95)	<0.001	<0.001	1.72 (1.56–1.90)	0.438 (0.282–0.571)	0.306 (0.169–0.430)
Normal weight vs. Obesity	0.99 (0.97–1.00)	<0.001	<0.001	2.58 (2.31–2.88)	0.099 (0.027–0.202)	0.072 (0.010–0.146)
Overweight vs. Obesity	0.88 (0.79–0.97)	<0.001	0.002	1.50 (1.32–1.70)	0.488 (0.314–0.637)	0.405 (0.250–0.538)
P3–P50 vs. P50–P85 †	0.66 (0.53–0.80)	0.019	0.125	1.16 (1.04–1.31)	0.757 (0.531–0.878)	0.708 (0.457–0.803)

$\hat{p}$, probability of superiority (probability that a randomly selected child from the second category has a larger *Σ*6ST than one from the first); *p*_adj_, Holm-adjusted *p*-value; GM, geometric mean from log-linear models adjusted for decimal age with HC3 standard errors; OVL, overlap coefficient with 95% bootstrap confidence interval; age-standardised OVL, computed from *Σ*6ST standardised to the mean age of the sample. † Secondary analysis within the normal-weight range; Holm adjustment across the six comparisons of the four-category scheme.

**Table 7:** Association of each skinfold with the sum of the remaining five skinfolds, individual prediction error for *Σ*6ST, and information added beyond BMI-for-age in boys and girls aged 6–12 year.

Skinfold Site	*ρ* with Remaining Five (95% CI)	MdAPE for *Σ*6ST (%)	95% LoA, Measured/Predicted	Δ*R*^2^ Beyond BMI *z* and Age	Partial *ρ* (95% CI)
**Boys**					
Triceps	0.935 (0.908–0.950)	9.5	0.74–1.32	0.169	0.819 (0.720–0.874)
Subscapular	0.931 (0.896–0.951)	12.7	0.70–1.38	0.122	0.741 (0.632–0.817)
Supraspinale	0.945 (0.915–0.961)	10.1	0.76–1.30	0.162	0.806 (0.714–0.868)
Abdominal	0.934 (0.903–0.952)	8.5	0.78–1.26	0.172	0.780 (0.689–0.836)
Thigh	0.867 (0.796–0.909)	14.1	0.65–1.46	0.107	0.721 (0.593–0.805)
Calf	0.906 (0.867–0.930)	11.0	0.72–1.36	0.135	0.696 (0.582–0.780)
*Reference* *: BMI z-score + age*	–	14.9	0.61–1.56	–	–
**Girls**					
Triceps	0.916 (0.851–0.948)	9.3	0.75–1.30	0.083	0.652 (0.482–0.780)
Subscapular	0.901 (0.806–0.948)	10.8	0.73–1.33	0.073	0.569 (0.366–0.753)
Supraspinale	0.901 (0.822–0.945)	9.1	0.75–1.30	0.076	0.583 (0.379–0.747)
Abdominal	0.913 (0.836–0.950)	9.5	0.75–1.31	0.070	0.606 (0.382–0.781)
Thigh	0.777 (0.622–0.885)	11.4	0.66–1.46	0.045	0.402 (0.121–0.648)
Calf	0.873 (0.776–0.929)	9.9	0.75–1.31	0.083	0.578 (0.322–0.747)
*Reference: BMI z-score + age*	–	11.5	0.69–1.41	–	–

*ρ*, Spearman rank correlation coefficient (all *p* < 0.001); CI, confidence interval; MdAPE, median absolute percentage error of leave-one-out predictions of *Σ*6ST from the single site (log–log model); LoA, limits of agreement for the ratio of measured to predicted *Σ*6ST; Δ*R*^2^, increase in explained variance of the log sum of the remaining five sites when the single site is added to BMI-for-age *z*-score and age (all *F*-test *p* < 0.001); partial *ρ*, controlling for BMI-for-age *z*-score and age.

**Table 8:** Zero-order and adjusted associations between right handgrip strength and anthropometric variables in boys and girls aged 6–12 years.

Variable	Zero-Order *ρ* (95% CI)	Partial *ρ* (95% CI) ‡	Allometric Elasticity (95% CI) §	Δ*R*^2^ §
**Boys**				
Body mass	0.654 (0.542 to 0.749)	–	–	–
Stature	0.723 (0.612 to 0.803)	–	–	–
Decimal age	0.611 (0.469 to 0.727)	–	–	–
Corrected arm girth	0.599 (0.473 to 0.696)	0.346 (0.153 to 0.505)	1.25 (0.66 to 1.84)	0.058
Forearm girth	0.576 (0.447 to 0.681)	0.239 (0.062 to 0.409)	1.05 (0.24 to 1.87)	0.027
Relaxed arm girth	0.453 (0.307 to 0.574)	−0.017 (−0.216 to 0.189)	0.38 (−0.46 to 1.21)	0.003
*Difference, corrected − relaxed arm girth*	0.146 (0.073 to 0.225)	0.364 (0.169 to 0.542)	–	–
**Girls**				
Body mass	0.759 (0.611 to 0.859)	–	–	–
Stature	0.683 (0.513 to 0.802)	–	–	–
Decimal age	0.606 (0.409 to 0.747)	–	–	–
Corrected arm girth	0.747 (0.584 to 0.862)	0.336 (0.081 to 0.626)	0.88 (−0.15 to 1.92)	0.032
Forearm girth	0.701 (0.534 to 0.821)	0.214 (−0.054 to 0.460)	0.90 (−0.39 to 2.19)	0.009
Relaxed arm girth	0.643 (0.459 to 0.775)	0.078 (−0.182 to 0.361)	0.10 (−1.00 to 1.20)	0.000
*Difference, corrected − relaxed arm girth*	0.104 (0.020 to 0.199)	0.258 (0.059 to 0.475)	–	–

*ρ*, Spearman rank correlation coefficient (all zero-order *p* < 0.001); CI, confidence interval; ‡, controlling for age, body mass and stature; §, coefficient of the log girth when added to a model of log handgrip strength on log body mass, log stature and age; Δ*R*^2^, increase in explained variance. Differences between dependent correlations were estimated with a paired bootstrap (5000 resamples).

## Data Availability

The datasets presented in this study can be found in online repositories. The names of the repositories and the accession numbers can be found at https://doi.org/10.6084/m9.figshare.33292314.

## Abbreviations

The following abbreviations are used in this manuscript:

BMI	Body mass index
CI	Confidence interval
IQR	Interquartile range
ISAK	International Society for the Advancement of Kinanthropometry
OVL	Overlap coefficient
STROBE	Strengthening the Reporting of Observational Studies in Epidemiology
*Σ*6ST	Sum of six skinfold thicknesses
WHO	World Health Organization
DXA	Dual-energy X-ray absorptiometry
GM	Geometric mean
HC3	Heteroscedasticity-consistent standard error, type 3
LoA	Limits of agreement
MdAPE	Median absolute percentage error
$\hat{p}$	Probability of superiority
